# Facile Automated Radiosynthesis of an Arginine Selective Bioconjugation Reagent 4‑[^18^F]Fluorophenylglyoxal for Developing Protein-Based PET Molecular Probes

**DOI:** 10.1021/acsomega.5c01278

**Published:** 2025-05-15

**Authors:** Pragalath Sadasivam, Shivashankar Khanapur, Siddesh V Hartimath, Boominathan Ramasamy, Peter Cheng, Chin Zan Feng, David Green, Julian L Goggi, Edward G Robins, Ran Yan

**Affiliations:** † Department of Imaging Chemistry and Biology, School of Biomedical Engineering and Imaging Sciences, King’s College London, London WC2R 2LS, U.K.; ‡ Institute of Bioengineering and Bioimaging, 54759 Agency for Science, Technology, and Research (A* STAR), 11 Biopolis Way, #01-02 Helios, Singapore 138667, Singapore; § Clinical Imaging Research Centre, #B01-01 Centre for Translational Medicine, Yong Loo Lin School of Medicine, National University of Singapore, 14 Medical Drive, Singapore 117599, Singapore; ∥ Molecular Imaging and Therapy Research Unit, 367695 South Australian Health and Medical Research Institute (SAHMRI), North Terrace, Adelaide, SA 5000, Australia; ⊥ Adelaide Medical School, Faculty of Health and Medical Sciences, University of Adelaide, North Terrace & George Street, Adelaide, SA 5000, Australia; # Minerva Imaging ApS, Lysho̷jvej 21, 3650 Ølstykke, Denmark; ∇ Department of Radiology and Medical Imaging, University of Virginia, Charlottesville, Virginia 22903, United States; ○ Radiochemistry Core, University of Virginia School of Medicine, Charlottesville, Virginia 22903, United States

## Abstract

4-[^18^F]­Fluorophenylglyoxal ([^18^F]­FPG) is a novel arginine selective bioconjugation reagent for native protein ^18^F-labeling. Here, we report the automated radiosynthesis of [^18^F]­FPG on a Scintomics GRP module. The radiochemical preparation was performed in a one-pot, two-step process using a DMSO-resistant cassette system. A cartridge-based purification method was developed to purify [^18^F]­FPG without HPLC. The [^18^F]­FPG was prepared in nondecay corrected (n.d.c.) radiochemical yields (RCYs) of 27 ± 2% (*n* = 5) in 56 min from the end of the bombardment until formulation. The molar activities of [^18^F]­FPG were 147 ± 70 GBq/μmol (*n* = 5). The 4-[^18^F]­FPG was then conjugated with interleukin-4 (IL-4) in n.d.c. 26 ± 2% RCYs (*n* = 3) from [^18^F]­FPG with molar activities of 24 ± 4 GBq/μmol (*n* = 3). [^18^F]­FPG-IL4 exhibited >95% stability in either PBS (4 h) or human serum (2 h) in vitro. [^18^F]­FPG-IL4 showed specific uptake by the PHA-activated Jurkat cells. The in vivo biodistribution and pharmacokinetics of [^18^F]­FPG-IL4 were evaluated in healthy Balb/c mice with PET imaging.

## Graphical abstract

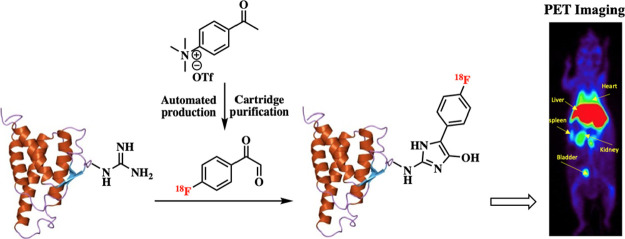

## Introduction

Positron Emission Tomography (PET) is a noninvasive molecular imaging technique widely used in nuclear medicine to diagnose, assess, and monitor various diseases.^1^ It enables the visualization of biological processes at the molecular level, providing valuable insights into disease progression and treatment response. In recent years, the development of protein-based PET molecular probes for diagnostics has gained increasing attention.^2^ Among them, the fluorine-18 labeled small proteins offer excellent targeting affinity, specificity, and selectivity. However, the harsh conditions typically required for direct radiolabeling with fluorine-18, such as elevated temperatures, organic solvents, and strong bases, are incompatible with sensitive biomolecules like proteins.^2^ To overcome this challenge, the use of ^18^F-prosthetic groups for protein labeling has become essential. Ideal prosthetic groups for fluorine-18 incorporation are defined by their low molecular weight, as well as their mild and rapid bioconjugation properties, ensuring compatibility with delicate biomolecules and preserving their structural and functional integrity.^3^ Several commonly used prosthetic groups targeting amine, aminooxy, thiol, and tyrosine, along with a newly developed arginine selective bioconjugation reagent, 4-[^18^F]­fluorophenylglyoxal ([^18^F]­FPG), are summarized in Figure 1.^4–6^ Among these, [^18^F]­FPG has been proven effective in radiolabeling of various small proteins.^6^ We successfully demonstrated [^18^F]­FPG labeled human serum albumin (HSA), [^18^F]­FPG-HSA for blood pool PET imaging as well as interleukin-2, [^18^F]­FPG-IL2 for monitoring T-cell infiltration in immune checkpoint therapy.^7^

**Figure 1:**
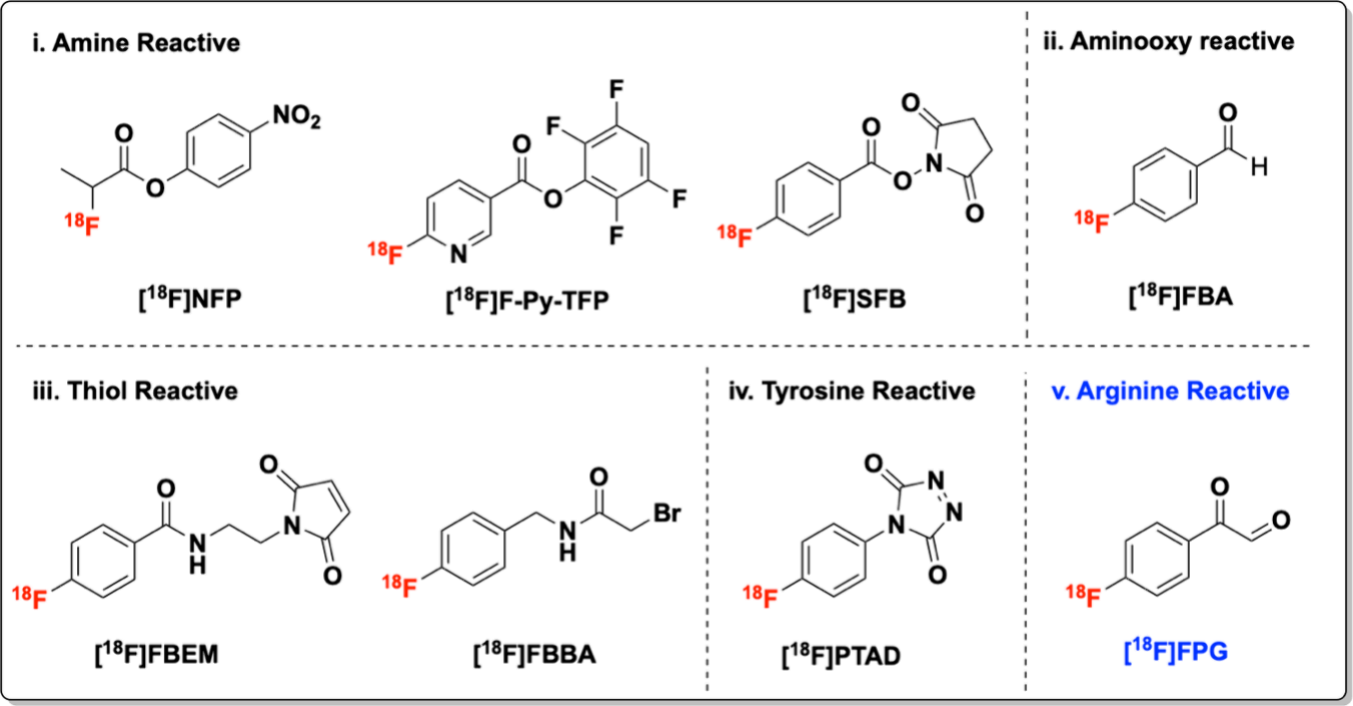
^18^F-prosthetic groups for protein bioconjugation.

To implement the clinical translation of [^18^F]­FPG labeled proteins, herein, we present the automated radiosynthesis and cartridge purification of [^18^F]­FPG on a Scintomics GRP module. We further demonstrated the facile radiolabeling of human interleukin-4 (IL-4) with [^18^F]­FPG to yield the [^18^F]­FPG-IL-4 bioconjugate. A preliminary in vitro and in vivo evaluation of [^18^F]­FPG-IL-4 was conducted to assess its uptake by the activated Jurkat cells and its biodistribution in healthy mice with PET imaging. These findings will serve as a foundation for future studies aimed at developing PET-based CAR T-cell imaging strategies.

## Results and Discussion

The radiosynthesis of [^18^F]­FPG is a one-pot, two-step process. (Scheme 1) First, the 4-acetyl-*N*,*N*,*N*-trimethylbenzenammonium triflate was reacted with fluoride-18 via a nucleophilic aromatic substitution chemistry to generate the intermediate, 4-[^18^F]­fluoroacetophenone ([^18^F]­FAcPh). Subsequently, [^18^F]­FAcPh was converted to [^18^F]­FPG by an I_2_/DMSO oxidation system in the same reaction vial. To develop a cartridge-based purification method, [^18^F]­FPG was prepared manually and then tested for trapping and releasing with an OASIS HLB Plus cartridge. It was found that about 91 ± 5% of [^18^F]­FPG in 13% DMSO in water can be trapped on the OASIS HLB Plus cartridge (*n* = 5). When eluting the OASIS HLB Plus cartridge with DMSO (1 mL), about 95 ± 2% (*n* = 5) of [^18^F]­FPG can be released. Thus, we decided to use the OASIS HLB Plus cartridge to purify [^18^F]­FPG in its automated radiosynthesis protocol. Next, we equipped the Scintomics GRP4 V synthesis module with the single-use, DMSO-resistant hardware kits. The cassette parts were acquired from ABX and assembled in-house. All the reagents were assembled for [^18^F]­FPG preparation, as illustrated in Figure 2A. The schematic diagram of the Scintomics GRP visualization file for the automated radiosynthesis of [^18^F]­FPG is demonstrated in Figure 2B. The assembly list of the [^18^F]­FPG radiosynthesis setup, together with the valve orientations in the Scintomics GRP4 V synthesis module, is summarized in Table 1.

**Scheme 1:**
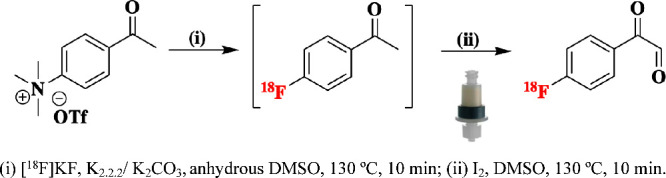
One-Pot Two-Step Automated Radiosynthesis and Cartridge Purification of [^18^F]­FPG; (i) [^18^F]­KF, K_2.2.2_/K_2_CO_3_, Anhydrous DMSO, 130 °C, 10 min; (ii) I_2_, DMSO, 130 °C, 10 min

**Figure 2:**
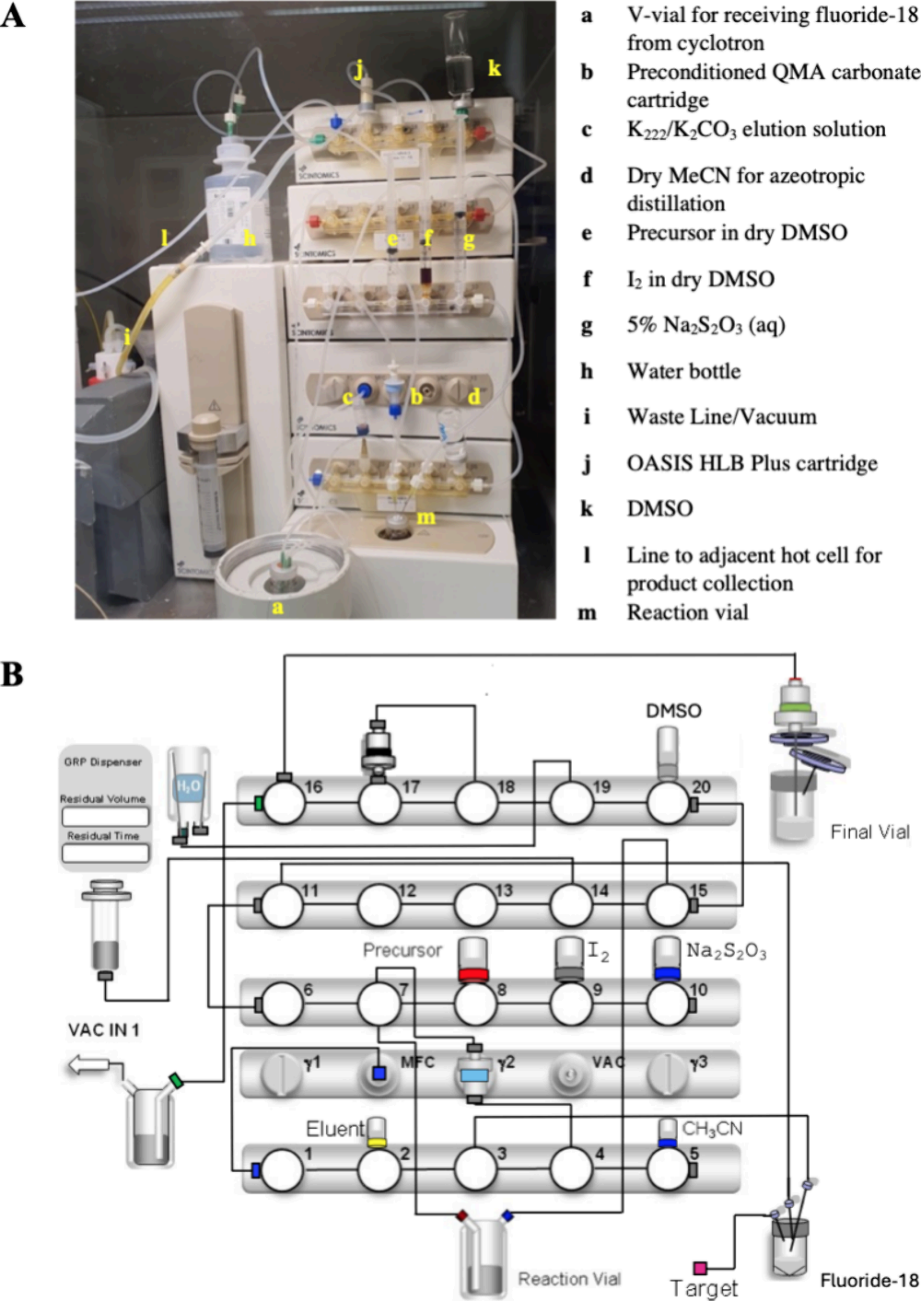
(A) Set-up of the Scintomics GRP4 V synthesis module with reagents for automated radiosynthesis of [^18^F]­FPG; (B) schematic diagram of the Scintomics GRP4 V synthesis module for automated radiosynthesis of [^18^F]­FPG.

**Table 1:** Assembly List of the [^18^F]­FPG Radiosynthesis Set-Up in a Scintomics GRP4 V Synthesis Module^a^

position	materials/reagents	details
1 H	Connection to a mass flow controller	
2 V	K_222_/K_2_CO_3_ eluent	K_222_/K_2_CO_3_ (5 mg/1 mg) in MeCN/H_2_O (0.8 mL/0.2 mL)
3 V	Tubing to V-Vial, inlet for F-18 (aq.)	
4 V	To QMA cartridge (female)	
5 V	Dry MeCN	5 mL
5 H	Tubing to bench 2, valve 10 H	
6 H	Tubing to bench 3, valve 11 H	
6 V	To QMA cartridge (male)	
7 V	To reaction vessel’s main port	
8 V	Precursor (5 mg) in dry DMSO (0.5 mL)	3 mL BD luer-lock syringe
9 V	I_2_ (25 mg) in dry DMSO (1 mL)	3 mL BD luer-lock syringe
10 V	5% Na_2_S_2_O_3_ (3 mL)	3 mL BD luer-lock syringe
11 H	Tubing to bench 2, valve 6 H	
11 V	Ventilation for V-vial	
12 V	Closed	
13 V	Closed	
14 V	Connection to syringe pump	20 mL BD luer-lock syringe
15 V	To reaction vessel ventilation port	
15 H	Tubing to bench 4, 20 H	
16 H	To the waste bottle	
16 V	To the product collection vial	In adjacent hot cell
17 V	OASIS HLB Plus cartridge	
18 V	To OASIS HLB Plus cartridge (female)	
19 V	To water bottle	100 mL deionized water
20 V	Product elution vial	1 mL DMSO
20 H	Tubing to bench 3, 15 H	

a V, vertical connection; H, horizontal connection.

Prior to the actual automated radiosynthesis, [^18^F]­fluoride (∼110 MBq) in H_2_O (3 mL) was transferred, trapped in a preactivated QMA cartridge and then eluted with K_222_/K_2_CO_3_ in MeCN/water (1 mL) to the reaction vial. Around 97% of activity was received in the reaction vial, which demonstrated the high fluoride-18 transfer and purification efficiency of the automated system. Next, five [^18^F]­FPG automated preparation runs were conducted, starting with (20–25 GBq) of [^18^F]­fluoride. The results are summarized in Table 2. [^18^F]­FPG was produced in ∼6 GBq in an average nondecay corrected (n.d.c.) radiochemical yield (RCY) of 27% within one hour. The molar activities were 147 ± 70 GBq/μmol (*n* = 5) with the radiochemical purity (RCP) >95% (Figure 3A). The identity of [^18^F]­FPG was confirmed by the coelution of its commercial nonradioactive reference compound. (Figure 3B) Compared to the manual radiosynthesis of [^18^F]­FPG (RCY: ∼41%),^6^ the automated radiosynthesis exhibited a reduced RCY of approximately 14%. This reduction is likely due to product and reagent losses occurring in the cassette kit, which results in lower overall RCYs. The [^18^F]­FPG, being a lipophilic molecule with a log *D* of ∼1.61,^6^ is more prone to losses during the automated transfer process. However, the automated production scaling up of [^18^F]­FPG to ∼6 GBq would not only improve the absolute activity available for downstream bioconjugation but also mitigate the radiation risks associated with manual radiosynthesis, enhancing both efficiency and safety. It also aligns the [^18^F]­FPG production with the Good Manufacturing Practice (GMP) regulations.^8^

**Table 2:** Summary of the [^18^F]­FPG Automated Radiosynthesis^a^ ^,^ ^b^

production	no.1	no.2	no.3	no.4	no.5
Fluoride-18	23.2 GBq	20.6 GBq	21.0 GBq	25.2 GBq	22.8 GBq
[^18^F]FPG	6.2 GBq	6.1 GBq	5.8 GBq	6.0 GBq	5.7 GBq
Duration	∼56 min				
% RCYs	26.7	29.6	27.6	23.8	25.0
Molar activity^a^	189.9	82.8	52.4	169.8	240.8
%RCP^b^	>95%				

a GBq/μmol.

b Determined by radioHPLC.

**Figure 3:**
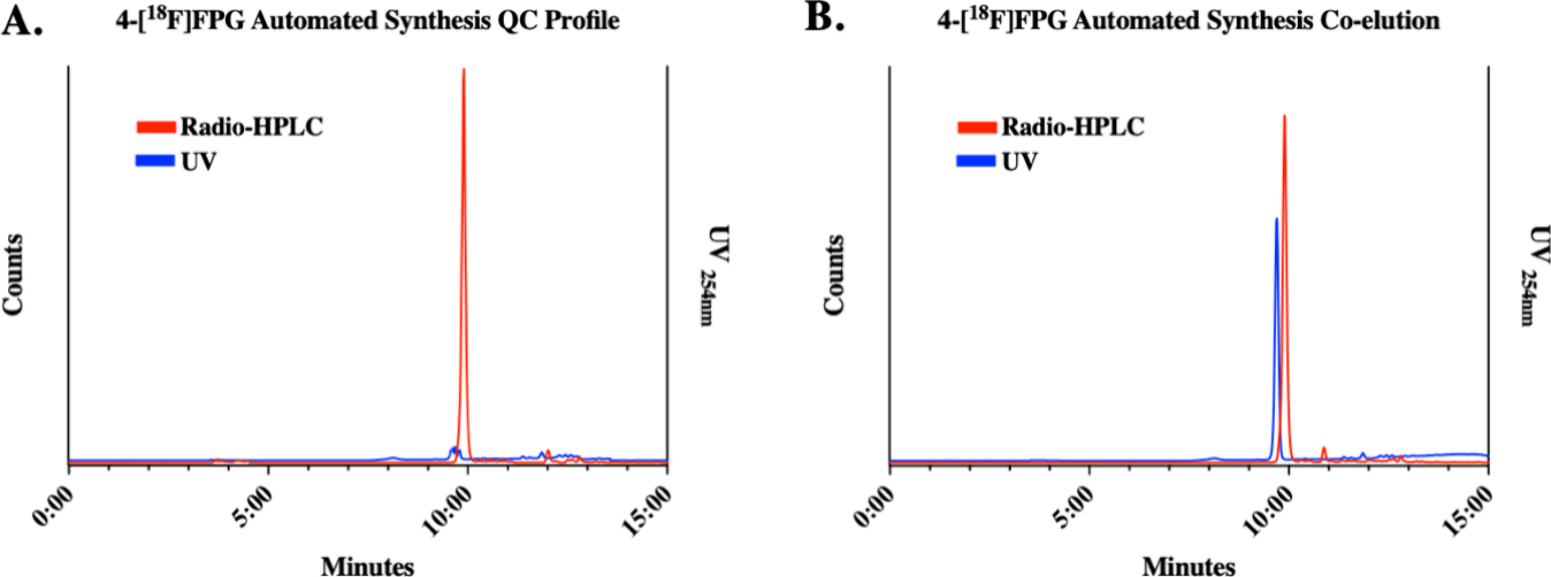
(A) Quality control of the cartridge purified [^18^F]­FPG; (B) co-elution of [^18^F]­FPG with its nonradioactive reference compound.

### Bioconjugation of [^18^F]­FPG with IL-4

To test the ability of the automatically produced cartridge purified [^18^F]­FPG for bioconjugation, we manually labeled IL-4 with [^18^F]­FPG in pH 9 phosphate buffer at 37 °C in 15 min. After size exclusion PD-10 column purification, [^18^F]­FPG-IL-4 was obtained in 26 ± 2% n.d.c. RCYs (*n* = 3) from [^18^F]­FPG. These RCYs are similar to that of the HPLC purified [^18^F]­FPG labeling of IL_4 (∼28%) reported previously,^6^ indicating the effectiveness of the cartridge purification process. The RCP of [^18^F]­FPG-IL-4 was >99% with the molar activities of 24 ± 4 GBq/μmol (*n* = 3). (Figure S1) Around 260 MBq of [^18^F]­FPG-IL-4 was manually produced starting with ∼1 GBq of [^18^F]­FPG, which is a clinically relevant quantity.

### In Vitro Stability of [^18^F]­FPG-IL-4

Subsequently, the in vitro stability of [^18^F]­FPG-IL-4 was determined in either 7.4 phosphate-buffered saline (PBS) or human serum at 37 °C. [^18^F]­FPG-IL-4 was found to be >95% stable in PBS for 4 h, which indicates that multiple patients could be scanned using a single batch of [^18^F]­FPG-IL-4. (Figure 4A) Moreover, [^18^F]­FPG-IL-4 was also stable in human serum for 2 h. (Figure 4B) The HPLC retention of the [^18^F]­FPG-IL-4 posthuman serum incubation was changed to a later time point, which might result from the interaction of [^18^F]­FPG-IL-4 with the human serum proteins as the entire incubation solution was injected into the HPLC.

**Figure 4:**
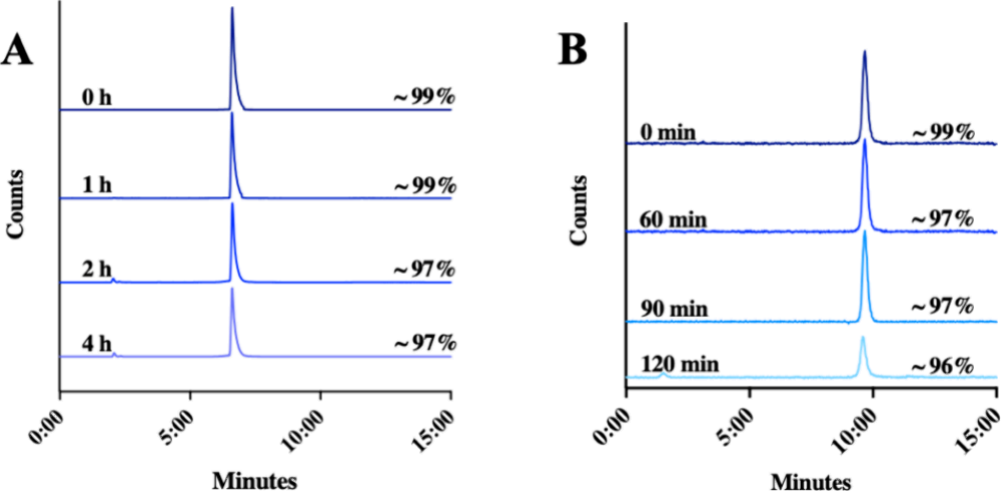
Stability of [^18^F]­FPG-IL-4 determined by HPLC in (A) PBS and (B) human serum.

To investigate the structural integrity of [^18^F]­FPG-IL-4, we performed SDS-PAGE analysis including native IL-4, purified [^18^F]­FPG-IL-4, and [^18^F]­FPG-IL-4 after 4 h of incubation in PBS. (Figure 5.) In both samples of [^18^F]­FPG-IL-4, only a single protein band was observed, matching the molecular size of native IL-4. These results indicate that [^18^F]­FPG conjugation preserves the structural integrity of IL-4, without causing protein degradation or aggregation. Moreover, it gave further evidence that [^18^F]­FPG-IL-4 remains stable in PBS in vitro. These data gave us the confidence to conduct the in vivo biological evaluation of [^18^F]­FPG-IL-4.

**Figure 5:**
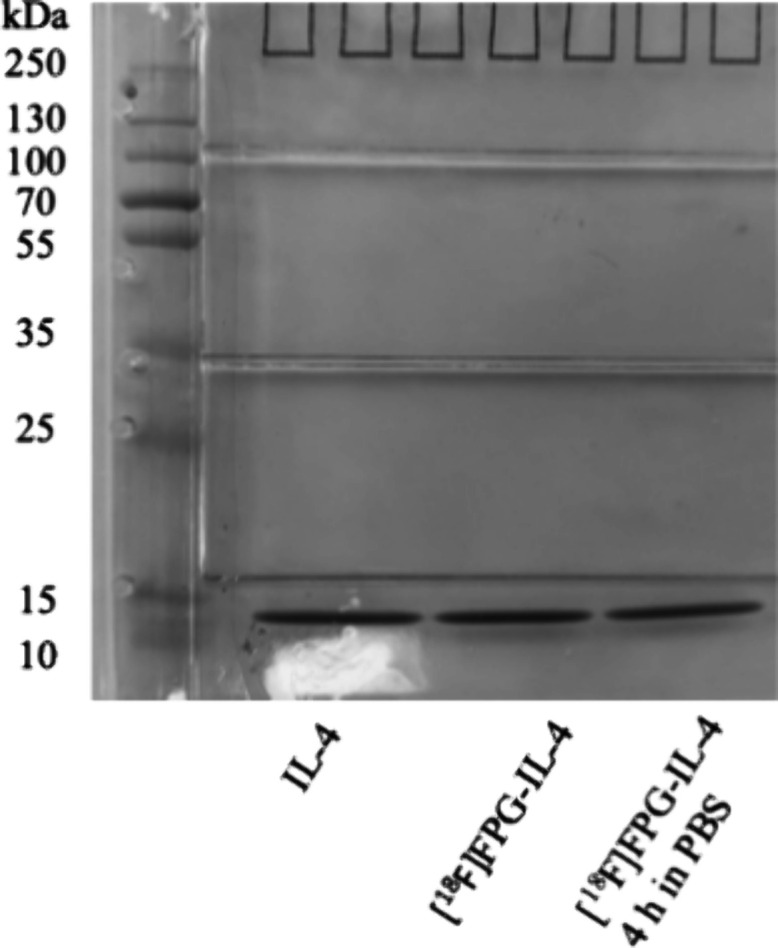
SDS-PAGE of native IL-4, purified [^18^F]­FPG-IL-4, and [^18^F]­FPG-IL-4 in PBS after 4 h.

### In Vitro Jurkat Cell Uptake of [^18^F]­FPG-IL-4

To investigate the binding of [^18^F]­FPG-IL-4 to the IL-4 receptors in T cells, the in vitro uptake of [^18^F]­FPG-IL-4 by the immortalized human T lymphocyte cells, Jurkat cells, was carried out. The phytohemagglutinin (PHA) activated and nonactivated Jurkat cells were incubated with [^18^F]­FPG-IL-4 at 37 °C for 30 min, respectively. The [^18^F]­FPG-IL-4 uptake by the activated and nonactivated Jurkat cells was 30.7 ± 0.4% per million cells and 4.4 ± 0.3% per million cells (*n* = 3), respectively. Blocking experiments were conducted by the pretreatment of the activated Jurkat cells with an excess of native IL-4 (400 ng/mL) for 30 min before [^18^F]­FPG-IL-4 addition. The Jurkat cell uptake of [^18^F]­FPG-IL-4 was reduced significantly to 7.4 ± 0.3% (****p < 0.0001, *n* = 3). (Figure 6) These data demonstrate that the activated Jurkat cell uptake of [^18^F]­FPG-IL-4 is IL-4 specific. Moreover, the *K*_d_ of [^18^F]­FPG-IL-4 was 0.273 nM, comparable to the *K*_d_ of native IL-4 of 0.105 nM, as reported previously,^6^ indicating that [^18^F]­FPG-IL-4 retains the binding affinity of native IL-4.

**Figure 6:**
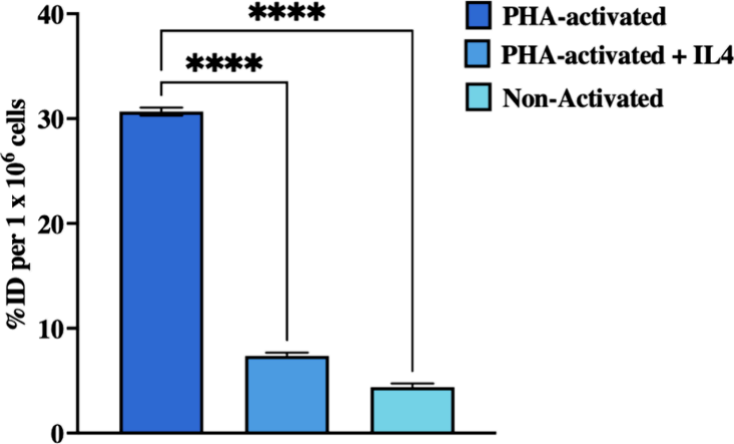
PHA-activated and nonactivated Jurkat cell uptake of [^18^F]­FPG-IL-4. Blocked samples were pretreated with 400 ng/mL native IL-4 prior to [^18^F]­FPG-IL-4 addition. Data shown are mean ± SD of three independent experiments conducted in triplicate (****P < 0.0001).

### PET Imaging and Ex Vivo Biodistribution Study of [^18^F]­FPG-IL-4

Finally, the pharmacokinetics and biodistribution of [^18^F]­FPG-IL-4 was evaluated in healthy Balb/C mice. The animals (*n* = 3) were subjected to 120 min dynamic PET imaging post intravenous (IV) injection of [^18^F]­FPG-IL-4. The radiotracer uptake was observed in major excretory organs, such as the liver and kidneys. (Figure 7A) The time–activity curves reveal a gradual decrease of radioactivity in the blood over 120 min. Meanwhile, rapid accumulation of radioactivity in the liver and kidneys was observed. Almost no [^18^F]­FPG-IL-4 uptake was observed in the brain, indicating that [^18^F]­FPG-IL-4 cannot penetrate the blood-brain barrier. Bone uptake was low and remained constant. This suggests that little in vivo defluorination took place. (Figure 7B).

**Figure 7:**
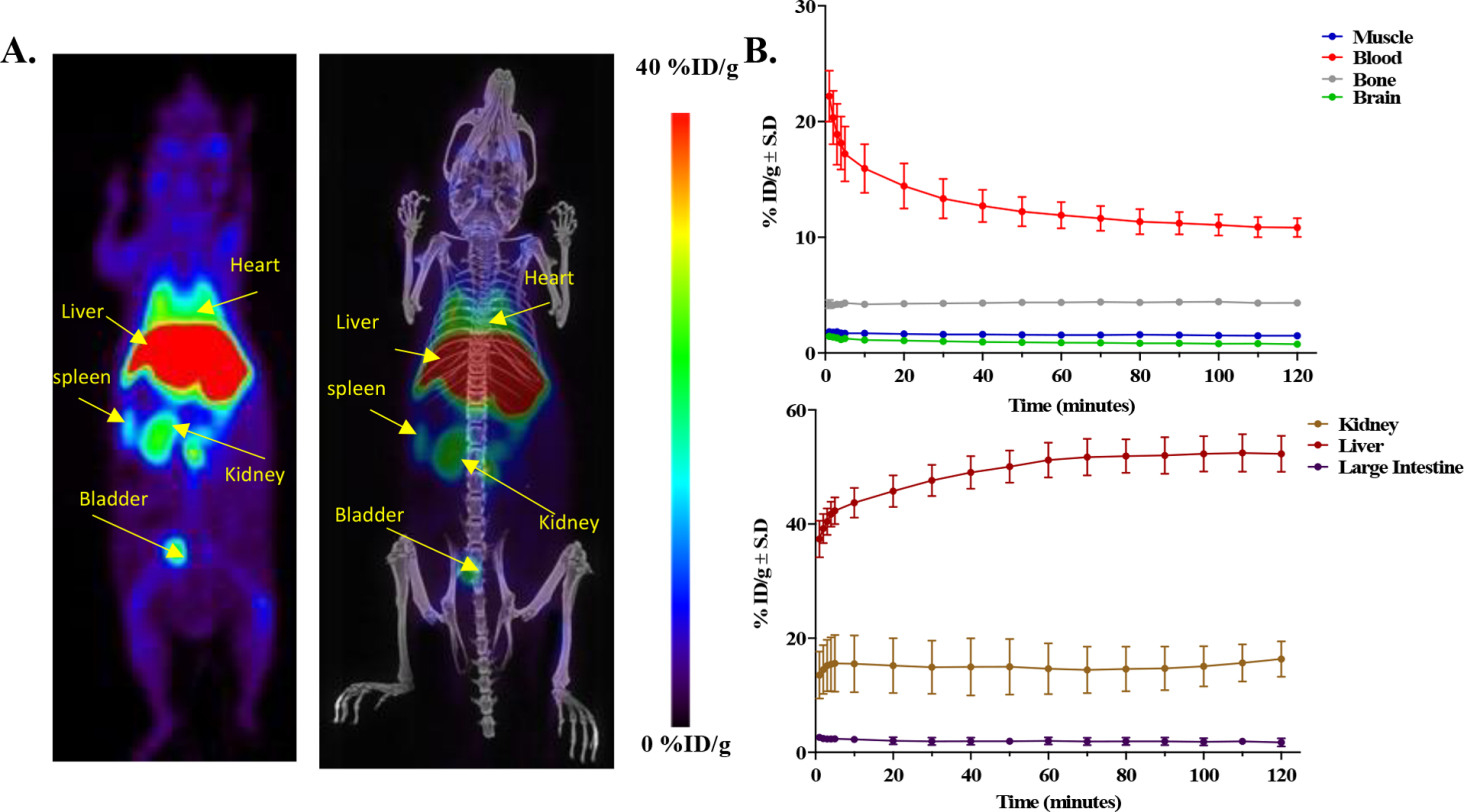
PET imaging of [^18^F]­FPG-IL-4 in healthy Balb/c mice. (A) Whole-body PET, PET, and CT fused images representing the summed frames from 0–120 min postinjection. (B) Dynamic time–activity curves showing uptake of [^18^F]­FPG-IL-4 in the major organs (*n* = 3).

The ex vivo biodistribution study of [^18^F]­FPG-IL-4 was also performed in healthy Balb/C mice at 30, 60, and 120 min post IV injection. The data were summarized in Figure 8 and Table S1. The radioactivity distribution is similar to the PET imaging, showing significantly higher uptake in the liver, kidney, and spleen while much lower uptake in other organs. The elevated liver and kidney uptake suggest hepatic and renal excretion pathways for [^18^F]­FPG-IL-4. Additionally, increased spleen accumulation of [^18^F]­FPG-IL-4 was observed. Given the elevated uptake of [^18^F]­FPG-IL-4 by the activated immortalized human T lymphocytes (Figure 6), we speculate that the higher spleen retention is likely due to T-cell-mediated uptake of [^18^F]­FPG-IL-4 within this organ. Both the [^18^F]­FPG-IL-4 PET imaging and its ex vivo biodistribution study give us the confidence to future develop [^18^F]­FPG-IL-4 for CAR T-cell in vivo tracking in the future.

**Figure 8:**
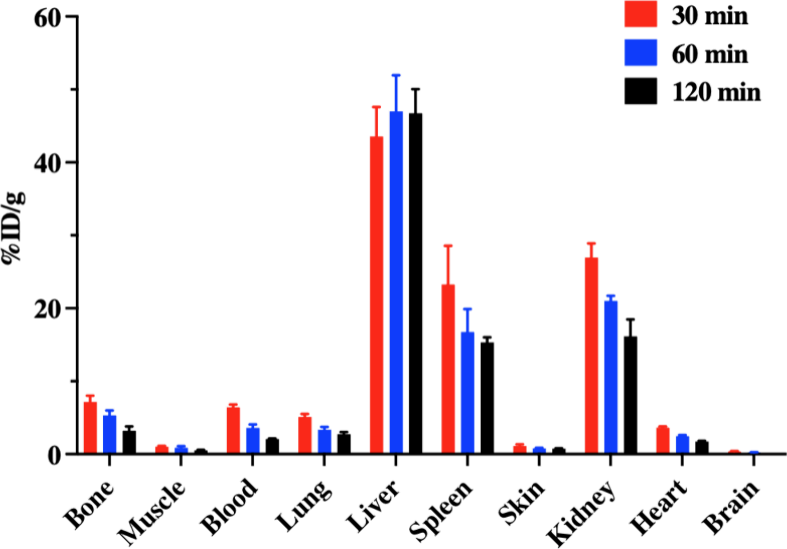
Biodistribution of [^18^F]­FPG-IL-4 in healthy Balb/c mice at 30, 60, or 120 min post IV injection (mean ± SD, *n* = 3, per time point).

## Conclusions

We have successfully developed an automated radiosynthesis and cartridge purification protocol for the arginine-selective prosthetic group, [^18^F]­FPG, using the Scintomics GRP modular lab. The system allows the production of [^18^F]­FPG in ∼6 GBq, with excellent radiochemical purity and high molar activity. The cartridge-purified [^18^F]­FPG was efficiently conjugated to IL-4, yielding sufficient [^18^F]­FPG-IL-4 for human PET imaging applications. Furthermore, the [^18^F]­FPG-IL-4 bioconjugate preserves the specificity and affinity toward the IL-4 receptor. PET imaging and biodistribution studies conducted in healthy Balb/c mice revealed that the [^18^F]­FPG-IL-4 conjugate is resistant to defluorination in vivo. These findings give us confidence that [^18^F]­FPG-labeled small proteins can be reliably produced in patient doses, paving the way for their applications in clinical PET imaging.

## Experimental Section

### General Information

4-Acetyl-*N*,*N*,*N*-trimethylbenzenammonium triflate was prepared according to previously published methods.^9,10^ Interleukin-4 (2 × 10^6^ IU), a recombinant interleukin-4 protein, was acquired from Miltenyi Biotec or Genscript, Germany and the United Kingdom, respectively. All reagents were procured from Sigma-Aldrich Pte Ltd., Singapore, Merck (Singapore), Tokyo Chemical Industry and Life Technologies Corporation (Singapore) and were used without further purification. Sep-Pak light (46 mg) Accell QMA carbonate cartridges and Oasis HLB Plus cartridges were purchased from Waters Pacific Pte Ltd., Singapore. PD-10 desalting columns were obtained from GE Healthcare Life Sciences, Singapore. No-carrier-added (n. c. a.) aqueous [^18^F]­fluoride was produced by the irradiation of ^18^O-enriched water via the [^18^O­(p, n)^18^F] nuclear reaction using a GE PETtrace 860 cyclotron (GE healthcare, Singapore). Co-elutions were performed on a Knaur semipreparative or UFLC Shimadzu radio-HPLC system (Shimadzu, Singapore). Radioactivity was measured with a CRC-55tPET dose calibrator (Capintec, Florham Park, NJ, USA).

### Automated Radiosynthesis of [^18^F]­FPG

At the end of the bombardment, aqueous fluoride-18 was transferred from the cyclotron into a V-vial through the vacuum. Upon complete transfer of activity, the radiosynthetic sequence commenced. The activity was drawn and trapped from the V-vial onto the QMA cartridge from valve 3 through valve 4. Aqueous fluoride-18 was eluted from the QMA cartridge using a solution of K_222_/K_2_CO_3_ (5 mg/1 mg) in MeCN/H_2_O (0.8 mL/0.2 mL) from valve 2 through valves 4 and 7 into the reaction vial. Removal of the solvents under vacuum at 115 °C proceeded for 5 min, followed by 3 × 1 mL of anhydrous MeCN added from valve 5, through valves 4 and 7 for azeotropic distillation to completely remove water. The precursor, 4-acetyl-*N*,*N*,*N*-trimethylbenzenammonium triflate (5 mg, 15 μmol) in anhydrous DMSO (500 μL) was added to the reaction vial from syringe 8 through valve 7. The reaction was heated at 130 °C for 10 min. I_2_ (25 mg, 10 μmol) in anhydrous DMSO (1 mL) was added to the reaction mixture from syringe 9 through valve 7. The reaction was heated at 130 °C for another 10 min. The crude reaction mixture was cooled to room temperature and quenched with 3 mL of 5% Na_2_S_2_O_3_ (*aq*) via syringe 10 through valve 7. The reaction mixture was then drawn into a 20 mL syringe, followed by an additional 10 mL of water from the water bag. The diluted mixture was passed through an OASIS HLB Plus cartridge. The cartridge was dried for 5 min under N_2_ flow. The [^18^F]­FPG was eluted from the cartridge with DMSO (1 mL) into a product collection vial.

### Quality Control of [^18^F]­FPG

The purified [^18^F]­FPG was analyzed via radio-HPLC. Analytical HPLC: Kinetex 5 μm F5 100 Å 150 × 4.6 mm. Mobile phase: 0.1% trifluoroacetic acid in both MeCN and H_2_O. Method: 5–95% MeCN from 0 to 7 min; 95% MeCN from 7 to 10 min; 95–5% MeCN from 10 to 15 min. Flow rate = 1.0 mL/min, λ = 254 nm. [^18^F]­FPG has an HPLC retention time of 9.55 min, **RCYs**: 27 ± 2%, **RCP**: >95%, **Molar activities**: 147 ± 70 GBq/μmol within 56 min. (*n* = 5, n.d.c.)

### Bioconjugation of [^18^F]­FPG with Interleukin-4

[^18^F]­FPG (∼1.0 GBq, *n* = 3) in DMSO (150 μL) was added to human IL-4 (13 nmol) in pH 7.4 PBS (850 μL). The reaction mixture was adjusted to pH ∼ 9.0 with TEA and incubated at 37 °C for 15 min at 500 rpm. The crude reaction mixture was purified via a PD-10 column and analyzed by radio-HPLC. Analytical HPLC: Jupiter 5 μm C4 300 Å 150 × 4.6 mm. Mobile phase: 0.1% trifluoroacetic acid in both MeCN and H_2_O. Method: 5–95% MeCN from 0 to 5 min; 95% MeCN from 5 to 6 min; 95–5% MeCN from 6 to 15 min. Flow rate = 1.0 mL/min, λ = 220 nm. [^18^F]­FPG-IL-4 has an HPLC retention time of 6.57 min, **RCYs**: 26 ± 2%, **RCP**: >99%, **Molar activities**: 24 ± 4 GBq/μmol. (*n* = 3, n.d.c.)

### In Vitro Stability Tests

#### In PBS

[^18^F]­FPG-IL-4 (∼1.0 MBq, *n* = 3) in pH 7.4 PBS was incubated at 37 °C and aliquots were taken at 0, 1, 2, and 4 h and analyzed via radio-HPLC. Analytical HPLC: Phenomenex Aeris Widepore 3.6 μm C4 300 Å 150 × 2.1 mm; Mobile phase: 0.1% formic acid in MeCN and H_2_O; Method: 5–95% MeCN from 0–5 min; 95% MeCN for 5–6 min; 95–5% MeCN for 6–15 min. Flow rate = 1.0 mL/min, *t*_R_ = 6.53 min.

#### In Human Serum

4-[^18^F]­FPG-IL-4 (∼1.0 MBq, *n* = 3) was incubated at 37 °C in human serum and aliquots were taken at 0, 60, 90, and 120 min and analyzed directly via radio-HPLC. Analytical HPLC: Phenomenex Aeris Widepore 3.6 μm C4 300 Å 150 × 2.1 mm Mobile phase: 0.1% formic acid in MeCN and H_2_O. Method: 5–95% MeCN from 0–5 min; 95% MeCN for 5–6 min; 95–5% MeCN for 6–15 min. Flow rate = 1.0 mL/min, *t*_R_ = 9.91 min.

### Sodium Dodecyl Sulfate Polyacrylamide Gel Electrophoresis (SDS-PAGE)

SDS-PAGE was conducted using a Bio-Rad Mini–PROTEAN3 system (BioRad Laboratories Ltd., UK. Tris-glycine gels (14%) were used. The gels were then covered in 1 L of 1× running buffer. A 250 kDa PageRule prestained protein ladder (2 μL, ThermoFisher, Loughborough, UK) was added into the first well of the gel. Native IL-4, purified [^18^F]­FPG-IL-4, and [^18^F]­FPG-IL-4 in PBS after 4 h (5 μg, each) were added to each well and then separated by electrophoresis. The gel was run at 140 V for 90 min, or until appropriate separation had occurred. The gel was then washed in PBS to remove any excess SDS before being submerged in Coomassie Blue protein stain and gently shaken for 20 min. The Coomassie blue stain was then discarded, and the gel was washed with destaining buffer until clear protein bands were visible.

### In Vitro Cell Uptake of [^18^F]­FPG-IL-4

Jurkat Clone E6–1 cells were cultured in RPMI-1640 medium supplemented with 5% fetal bovine serum and 1% Penicillin Streptomycin solution. Jurkat Clone E6–1 cells were either activated or nonactivated by incubation with or without 2 μg/mL of PHA (Sigma-Aldrich, Singapore), respectively, in a 5% CO_2_ atmosphere at 37 °C for 12 h. Approximately 1 × 10^5^ Jurkat Clone E6–1 cells were incubated with 200 KBq of [^18^F]­FPG-IL-4 at 37 °C for 30 min. (*n* = 3) After incubation, the Jurkat cells were washed with 1 mL ice-cold PBS and cell-bound activity was measured using a gamma counter. Blocking experiments were performed where the activated Jurkat cells were preincubated with IL-4 (400 ng/mL) at 4 °C for 30 min, prior to the addition of [^18^F]­FPG-IL-4. (*n* = 3)

### Preclinical Evaluation of [^18^F]­FPG-IL-4

All animal procedures were carried out in accordance with the Institutional Animal Care and Use Committee Singapore (IACUC No. 181399) and conformed to the US National Institutes of Health (NIH) guidelines and public law. Balb/c mice aged 6–8 weeks were purchased from In Vivos Singapore and kept at room temperature with a 12-h light-dark cycle and had free access to food and water.

### PET Imaging of [^18^F]­FPG-IL-4

Healthy Balb/c mice were anesthetized using a mixture of isoflurane and medical air (5% induction, 2% maintenance) and kept on electronic heating pads during the scanning period. Each animal was injected with 9 ± 2 MBq of [^18^F]­FPG-IL-4 via the lateral tail vein, and immediately, a dynamic PET scan of 120 min was performed. During the scanning, animals were monitored for body temperature and respiration rate using a Biovet physiological monitoring system. PET images were corrected for decay and scatter and iteratively reconstructed to 16 frames (5 × 60, 1 × 300, 10 × 600 s). The radioactive uptake in different organs was estimated by drawing a volume of interest (VOI) to generate the time-activity curves. The reconstructed calibrated images were analyzed using Amide software (version 10.3 Sourceforge). The data were expressed as a percentage of injected dose per gram (% ID/g) in the VOI.

### Ex Vivo Biodistribution of [^18^F]­FPG-IL-4

Healthy Balb/c mice (*n* = 3, per time point) were injected with [^18^F]­FPG-IL-4 (∼1.0 MBq) via the lateral tail vein and sacrificed at 30, 60, and 120 min postinjection. The major organs were excised and weighed. The radioactivity was quantified using a Wizard 2470 PerkinElmer gamma counter. The radioactivity uptake in the organs was expressed as the percentage of injected dose per gram of tissues (% ID/g).

## Supplementary Material



## Notes

Figure S1, HPLC chromatogram of purified [^18^F]­FPG-IL-4 quality control; and Table S1, biodistribution of [^18^F]­FPG-IL-4 in healthy Balb/c mice at 30, 60, or 120 min post IV injection (*n* = 3, per time point) (PDF)

## Abbreviations

**[** ^**18**^ **F]­FPG**: 4-[^18^F]­fluorophenylglyoxal
IL-4: interleukin-4
PBS: 0.1 M phosphate-buffered saline, pH 7.4
PET: positron emission tomography
