# Supplementary material for: Facile Automated Radiosynthesis of an Arginine Selective Bioconjugation Reagent 4‑[18F]Fluorophenylglyoxal for Developing Protein-Based PET Molecular Probes
Source: ACS Omega. 2025 May 15;10(20):20570–7. doi: 10.1021/acsomega.5c01278 (PMC12120570; doi:10.1021/acsomega.5c01278)
Supplement: Supplementary file 1 [file ao5c01278_si_001.pdf]

Supporting Information

Facile Automated Radiosynthesis of An Arginine Selective Bioconjugation Reagent 4-[^{18}F]Fluorophenylglyoxal for Developing Protein-based PET Molecular Probes

Authors: Pragalath Sadasivam[1,2,3,6], Shivashankar Khanapur[2,7,8], Siddesh V Hartimath[2], Boominathan Ramasamy[2], Peter Cheng[2], Chin Zan Feng[2], David Green[3], Julian L Goggi[2,6], Edward G Robins[2,3,4,5], Ran Yan[1*]

[1]School of Biomedical Engineering and Imaging Sciences, Department of Imaging Chemistry and Biology, King's College London, UK.

[2]Institute of Bioengineering and Bioimaging, Agency for Science, Technology, and Research (A* STAR), 11 Biopolis Way, #01-02 Helios, Singapore 138667, Singapore.

[3]Clinical Imaging Research Centre, 14 Medical Drive, #B01-01 Centre for Translational Medicine, Yong Loo Lin School of Medicine, National University of Singapore, Singapore 117599, Singapore.

[4]Molecular Imaging and Therapy Research Unit, South Australian Health, and Medical Research Institute (SAHMRI), North Terrace, Adelaide SA 5000, Australia.

[5]Adelaide Medical School, Faculty of Health and Medical Sciences, University of Adelaide, North Terrace & George Street, Adelaide SA 5000, Australia.

[6]Minerva Imaging ApS, Lyshøjvej 21, 3650 Ølstykke, Denmark.

[7]Department of Radiology and Medical Imaging, University of Virginia, Charlottesville, VA, USA.

[8]Radiochemistry Core, University of Virginia School of Medicine, Charlottesville, VA, USA.

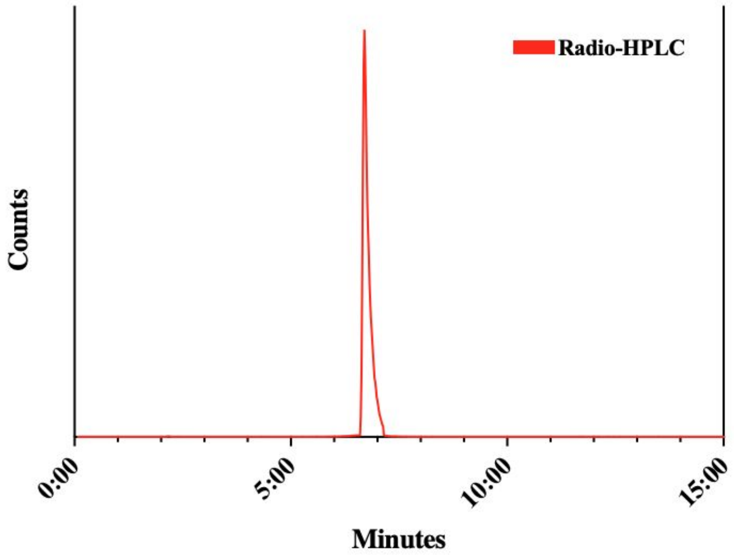

Figure S1. HPLC chromatogram of purified [^{18}F]FPG-IL-4 quality control.

Table 1S. Biodistribution of [^{18}F]FPG-IL-4 in healthy Balb/c mice at 30, 60, or 120 min post IV injection (mean±SD, n = 3, per timepoint).

Organ	30 min	60 min	120 min
Bone	7.17 ± 1.56	5.33 ± 1.15	3.23 ± 1.00
Muscle	1.03 ± 0.18	0.88 ± 0.35	0.53 ± 0.08
Blood	6.44 ± 0.67	3.60 ± 0.85	2.09 ± 0.08
Lung	5.08 ± 0.74	3.36 ± 0.62	2.75 ± 0.49
Liver	43.58 ± 6.94	46.99 ± 8.60	46.74 ± 5.74
Spleen	23.26 ± 9.2	16.75 ± 5.45	15.32 ± 1.22
Skin	1.14 ± 0.36	0.79 ± 0.13	0.76 ± 0.10
Kidney	26.96 ± 3.33	21.01 ± 1.21	16.15 ± 4.02
Heart	3.63 ± 0.28	2.47 ± 0.27	1.72 ± 0.21
Brain	0.40 ± 0.03	0.32 ± 0.05	0.13 ± 0.01
